# Identification of two immune subtypes and four hub immune-related genes in ovarian cancer through multiple analysis

**DOI:** 10.1097/MD.0000000000035246

**Published:** 2023-10-06

**Authors:** Qin Tang, Haojie Zhang, Rong Tang

**Affiliations:** a Department of Obstetrics and Gynecology, The Jingmen Center Hospital, Jingmen, PR China; b Department of Operating Room, The Jingmen Center Hospital, Jingmen, PR China; c Department of Pathology, The Jingmen Center Hospital, Jingmen, PR China.

**Keywords:** gene set enrichment analysis, immunotype, ovarian cancer, western blotting, WGCNA

## Abstract

Immune classification of ovarian cancer (OV) becomes more and more influential for its immunotherapy. However, current studies had few immune subtypes of OV. It is urgent to explore the immune subtypes and deeper hub immune-related genes (IRGs) of OV for follow-up treatment. A total number of 379 OV samples were obtained from UCSC online website. Single sample gene set enrichment analysis of 29 immune gene sets was used for identifying immune subtypes of OV and gene set variation analysis were used for exploring the hallmarks and Kyoto Encyclopedia of Genes and Genomes pathways of immune types. Two immunity subtypes (Immunity_H and Immunity_L) were identified by single sample gene set enrichment analysis. The OV patients in Immunity_H group had longer overall survival compared with those in Immunity_L group. The Immunity_H had higher stromal score, immune score and estimate score and the tumor purity had the adverse tendency. Besides, the gene set variation analysis enrichment results showed positive relationship between improved immunoreaction and pathways correlated to classical signaling pathway (PI3K/AKT/MTOR, P53, TNFA/NFkB signaling pathways) and immune responses (T/B cell receptor signaling pathways and primary immunodeficiency). Furthermore, 4 hub IRGs (CCR5, IL10RA, ITGAL and PTPRC) were jointly dug by weighted gene co-expression network construction and Cytoscape. Our team also explored the mutations of 4 hub IRGs and PTPRC showed nearly 7% amplification. Besides, 8 immune-checkpoint genes had higher expression in Immuity_H group compared with Immuity_L group, except CD276. The correlation between PD-1/PD-L1 and 4 hub IRGs were explored and gene set enrichment analysis were conducted to explore the underlying mechanisms of PTPRC in OV. Finally, western-blotting showed PTPRC could regulate immune checkpoint PD-L1 expression via JAK-STAT signaling pathway. In a word, 2 immune subtypes and 4 hub IRGs of OV were identified by multiple analysis.

## 1. Introduction

Ovarian cancer (OV) is one of the 3 major malignant tumors of female reproductive system, and its incidence is second only to cervical cancer and uterine body cancer, ranking third in female reproductive system malignant tumors. OV is a serious threat to women’s health in China due to its high degree of malignancy, high recurrence rate and difficult treatment.^[1]^ Debulking surgery with platinum-taxane maintenance chemotherapy is the principal treatment for OV.^[2]^ Although, the 5-year overall survival rate of OV has risen from 37% to 45%, the overall survival of advanced OV is merely 17%.^[3]^ Serous OV is one of the most common death types in OV, classified into low-grade and high-grade pathological type. Thus, it`s urgent for people to explore a novel and efficient way to treat OV. In the last decade, immune checkpoint inhibitors were widely applied for multiple cancers.^[4,5]^ Some malignant tumors, like non-small-cell lung cancer and melanoma had made an inspiring progress by immunotherapy.^[6,7]^ With the development of targeted therapy and immunotherapy, polyadenosine diphosphate ribose polymerase inhibitors have been developed and applied in maintenance therapy of OV, which has completely changed the plight of OV treatment. However, due to the complex immune landscape, immunotherapy has not been diffusely applied for OV.^[8]^ Previous studies indicated that immune procedures might play vital roles in evolution of OV.^[9,10]^ One research reported that a better clinical prognosis model related to the immune microenvironment was established for OV.^[11]^ Another study identified 10 immune microenvironment genes related to the prognosis of OV.^[12]^ Besides, 2 lncRNA-related molecular subtypes which had significant different prognosis and immunological characteristics by analyzing OV cohort in The Cancer Genome Atlas (TCGA).^[13]^ Recently, 1 research reported that exosomes might play a vital role in OV immunotherapy as an effective, rapid and safe carrier for delivery of ant-tumor drugs.^[14]^ Despite the increased interests in immunotherapy in OV, the mechanisms which the microenvironment cells regulate the tumorigenesis and progression for OV have not been widely probed and validated.

By analyzing TCGA-OV cohort based on immune gene sets, our team explored 2 obvious and stable immune subtypes. And the high immunity group had a long-term survival rate compared with low immunity group. After using Gene Co-expression Network Analysis and cytoHubba in Cytoscape, 4 hub immune-related genes (IRGs) (CCR5, IL10RA, ITGAL and PTPRC) were excavated. Furthermore, these IRGs were explored by searching cBioPortal website and PTPRC was found more amplification rather other hub IRGs. Also, we found the mutation site and protein expression of PTPRC. The high correlation between 4 IRGs and PD-1/PD-L1 and the GSEA results of PTPRC further emphasize these 4 IRGs promote progression of OV via multiple ways, especial immune related pathways. Finally, we proved that down-regulation of PTPRC could reduce the expression of PD-L1 through JAK-STAT signaling pathway by western-blotting. All these findings might provide a visionary insight for OV immunotherapy in the future.

## 2. Materials and methods

### 2.1. Data source and processing

Gene expression profile and corresponding survival information of a total number 379 TCGA-OV patients were downloaded from UCSC Xena website (https://xena.ucsc.edu/public). The gene symbol annotations were obtained from GENCODE website (https://www.gencodegenes.org/). The normalization of TCGA-OV gene expression was used log2 method.

### 2.2. Identification of OV subtypes based on the immune genes

Some studies indicated that 29 immune gene sets could represent tumor immunity.^[15,16]^ And GSVA package was used for single-sample gene set enrichment analysis (ssGSEA) of 29 immune gene sets for TCGA-OV.^[17]^ The “pheatmap” package was used for plotting heatmap. A clustering R package, ConsensusClusterPlus, was used to cluster and screen molecular subtypes according to immune gene expression profiles.^[18]^ The cumulative distribution function curve was used for selecting the most suitable cluster number.^[19]^ And principal component analysis was used to validate the credibility of the consensus cluster.^[20]^

### 2.3. Overall survival analysis and immune scores

To compare different immunity group overall survival, the “survival” and “survminer” packages were used for this assessment. To explore the TCGA-OV tumor cell infiltration and composition, the expression was calculated by ESTIMATE algorithm^[21]^ in R. The different situation in each group was calculated by Wilcoxon test.

### 2.4. Weighted gene co-expression network construction

To explore the concrete distribution of hub IRGs in each subtype of TCGA-OV and to discover high correlation modules with immune cell infiltration, our team downloaded 1792 IRGs from online website IMMPORT (https://www.immport.org/) and constructed an expression matrix by weighted gene co-expression network construction (WGCNA) package in R.^[22]^ With building an unsigned topological overlap matrix, an appropriate soft-thresholding power that would ensure scale-free topology R^2^ close to 0.9. The minimum number of IRGs in the module was 30, and the branch merge interception height was 0.25. This result was showed by a cluster dendrogram.

### 2.5. Significant modules and hub IRGs confirmation

The correlation between modules and clinical information was calculated in R. The highest correlation module was chosen for next analysis. Then, IRGs with high within-module connectivity were confirmed the hub IRGs (cor.geneModuleMembership > 0.9). To seek the real hub IRGs, we put this network into Cytoscape software.^[23]^ CytoHubba app was also used for selected hub IRGs.^[24]^ Two methods, including Degree and MCC, were used for selected top10 hub IRGs. Next, we intersected this 3 hub IRGs for the final hub IRGs.

### 2.6. Mutation and immunohistochemical information for IRGs

The cBioPortal online website was used for exploring the mutation of all IRGs.^[25]^ We chose PTPRC for subsequent analysis because of its high amplification. And the mutation site was also be plotted in this website. We also utilized The Human Protein Atlas (https://www.proteinatlas.org/) for mining the protein expression between ovary and OV.^[26]^

### 2.7. Correlation and gene set enrichment analysis

To explore the correlation between these 4 hub IRGs and PD-1/PD-L1, our team analyzed the correlation by utilizing TCGA-OV data. As for gene set enrichment analysis (GSEA) for PTPRC, after divided 2 groups (high and low expression group) by median value of PTPRC, we performed GSEA using GSEA software (Version 3.0). And the “gene sets database” was used “c2.cp.kegg.v7.4.” The *P* value < .05 was selected to presented in our results.

### 2.8. Experimental verification

To validate the influence of PTPRC on immune and JAK-STAT signaling pathway, the qPCR and western-blotting were conducted. Two kinds of OV cells, ES-2 and OVCAR3 were obtained from the Cell Bank of Type Culture Collection (CBTCC, the Chinese Academy of Sciences, Shanghai, China). All OV cells were cultured in DMEM (HyClone) with 10% fetal bovine serum (Gibco) at 37°C. The total RNA was extracted using PureLink^TM^ RNA (Thermo Fisher) from 2 OV cells and reverse transcription of mRNAs was operated using PrimeScript RT reagent Kit (Takara, Japan). Quantitative real-time PCR (q-PCR) was using by a CFX connect Real-time PCR detection system (Bio-Rad) with SYBR Green PCR kit (Toyobo, Japan). The action of qPCR was conducted by corresponding steps. All small interfering RNAs (siRNAs) for knocking down PTPRC were designed by GeneCreat (Wuhan GeneCreat Biological Engineering, Wuhan). All sequence of siRNAs were listed in Table S1, Supplemental Digital Content, http://links.lww.com/MD/J928. Lipofectamin 2000 reagent was used for transfecting. All OV cells protein extraction were used RIPA buffer after transfection 48 hour. The western-blotting was conducted according with corresponding steps. All antibody information was also listed in Table S1, Supplemental Digital Content, http://links.lww.com/MD/J928.

### 2.9. Statistical analysis

All statistical analysis were conducted by R (version 4.1.0). Data with 2 groups was analyzed by *t* test or Wilcoxon test. Overall survival information was analyzed by Cox and Kaplan–Meier method. *P* value < .05 was considered to be statistically significant.

## 3. Results

### 3.1. Two immune subtypes of OV were identified

**Figure 1.**
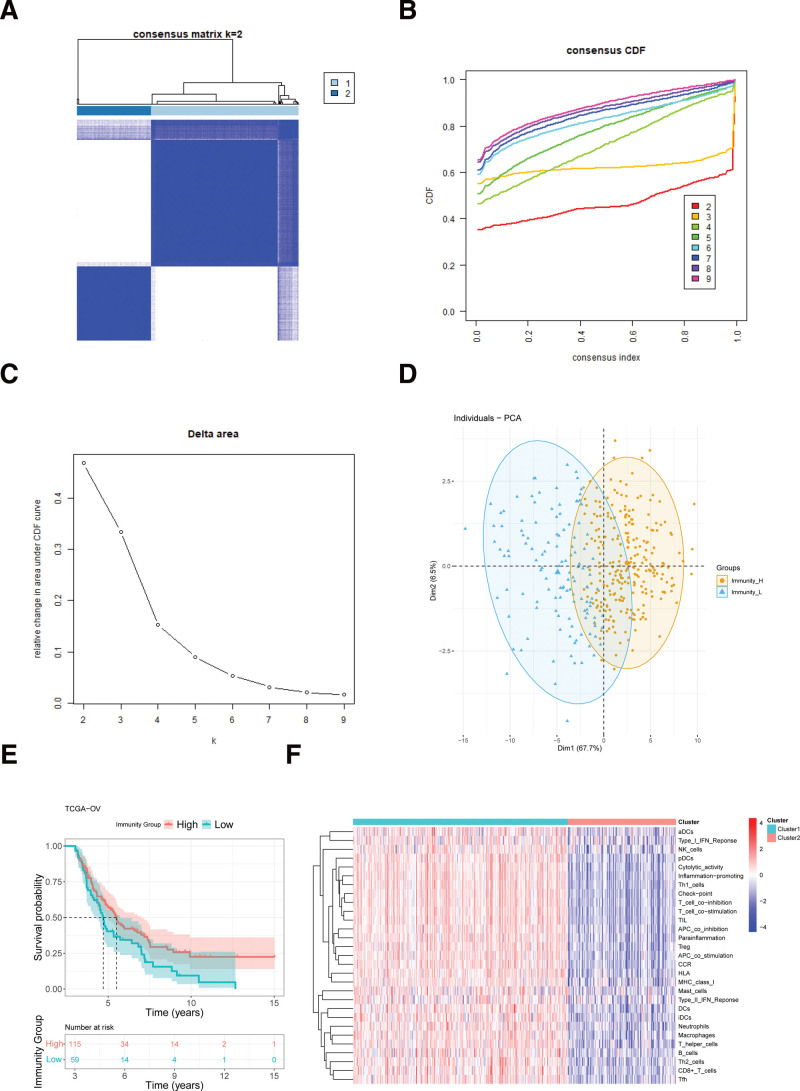
Consensus clustering for TCGA-OV datasets. (A) The consensus score matrix for OV samples when *k* = 2. A higher consensus score between 2 samples indicated that they were more likely to be distributed to the same cluster in different iterations. (B) The CDF curve of scores for 8 subtypes (*k* = 2–9). (C) Delta area curve of all OV samples. (D) PCA of all OV samples based on ssGSEA scores. (E) Overall survival of OV patients in 2 immune subtypes. High Immunity group indicated better long-term survival status compared with low Immunity group. (F) Heatmap of 2 clusters (Cluster1 represented Immunity_L and Cluster2 represented Immunity_H). CDF = cumulative distribution function, OV = ovarian cancer, PCA = principal component analysis, ssGSEA = Single sample gene set enrichment analysis, TCGA = The Cancer Genome Atlas.

All immune scores of each OV sample were calculated by the ssGSEA enrichment using 29 immune gene sets. Then, all OV samples were separated into different subtypes (*k* = 2–9) by Consensus Cluster Plus. All OV samples were identified to 2 subtypes (*k* = 2) based on the cumulative distribution function curve (Fig. 1A–C). Principal component analysis was also validated these 2 subtypes and these 2 groups (Immunity_H and Immunity_L) had rather distinction (Fig. 1D). Kaplan–Meier survival analysis showed that high immunity group had better long-term overall survival compared with low immunity group (Fig. 1E). And the heatmap showed significant difference in these 2 subtypes (Fig. 1F).

### 3.2. Immune characteristics in 2 immune subtypes

**Figure 2.**
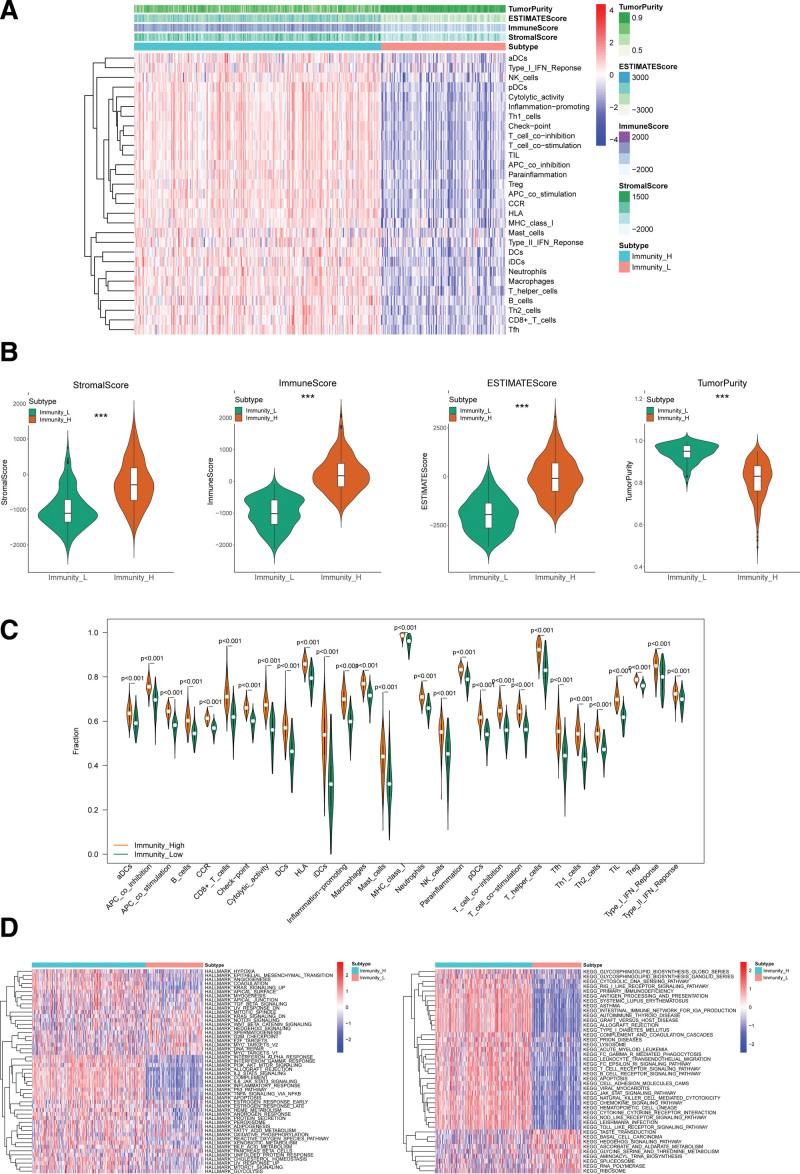
Identification of 2 immune subtypes in TCGA-OV dataset. (A) The heatmap of the 2 immune subtypes (Immunity_H and Immunity_L) based on ssGSEA scores for 29 immune gene sets. (B) The boxplot of stromal score, immune score, estimate score and tumor purity in 2 immune subtypes. (C) Express distribution of 29 immune gene sets in 2 immune subtypes. (D) The heatmap of enrichment scores of hallmarks and KEGG pathways in 2 immune subtypes by GSVA. GSVA = Gene Set Variation Analysis, KEGG = Kyoto Encyclopedia of Genes and Genomes, OV = ovarian cancer, ssGSEA = Single sample gene set enrichment analysis, TCGA = The Cancer Genome Atlas.

To explore deeper immune mechanisms of 2 immune subtypes, the stromal score, immune score, estimate score and tumor purity of each OV samples were calculated by the ESTIMATE algorithm. The heatmap showed expression tendency of 29 immune gene sets in each group (Fig. 2A). And the stromal score, immune score and estimate score had a higher expression in Immunity_H compared with Immunity_L. Nevertheless, tumor purity had the adverse tendency in these 2 groups (Fig. 2B). The violin plot also showed a higher expression tendency in Immunity_H compared with Immunity_L (Fig. 2C). Next, we conducted gene set variation analysis (GSVA) to explore the molecular pathways and mechanisms related to the immune subtypes of OV. And the GSVA enrichment results of OV indicated positive relationship between improved immunoreaction and pathways correlated to classical signaling pathway (PI3K/AKT/MTOR, P53, TNFA/NFkB signaling pathways) and immune responses (T/B cell receptor signaling pathways and primary immunodeficiency) (Fig. 2D).

### 3.3. Key module screening by WGCNA

**Figure 3.**
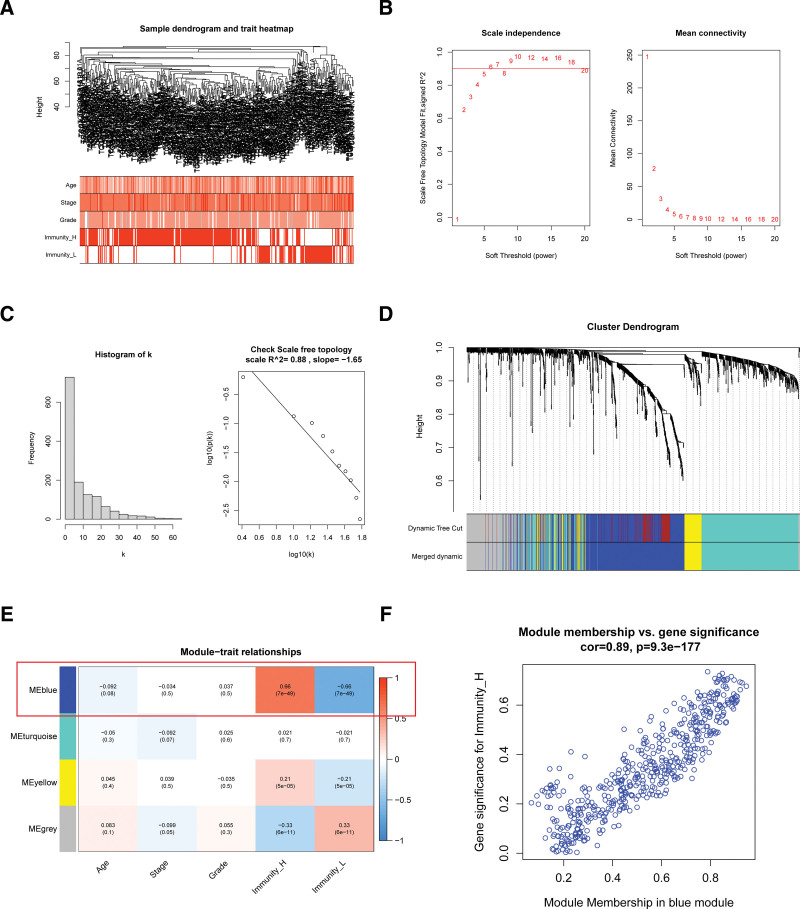
Identification of immune-related hub module of OV by WGCNA. (A) Samples dendrogram and trait heatmap. (B) Analysis of the scale-free fit index for various soft-thresholding powers (β) and the mean connectivity for various soft-thresholding powers. (C) Checking the scale free topology when β = 5. (D) Dendrogram of all differentially expressed genes clustered based on a dissimilarity measure. (E) Heatmap of the correlation between module eigengenes and clinical information of OV, and the MEblue showed 0.66 correlation with Immunity_H. (F) Module membership in blue module. OV = ovarian cancer, WGCNA = Weighted gene co-expression network analysis.

To identify hub IRGs in OV, WGCNA was used for the preliminary screening. All samples had no obvious outliers (Fig. 3A). We chose soft-thresholding β = 5 (scale-free *R*^2^ = 0.88, slope = −1.65) for constructing a scale-free network (Fig. 3B and C). A total of 4 modules, including MEblue, MEturquoise, ME yellow and MEgrey were identified by assembling similar IRGs (Fig. 3D). And the IRGs in blue module had a high correlation (cor = 0.66, *P* value = 7e-49 and cor = −0.66, *P* value = 7e-49) with Immunity_H and Immunity_L (Fig. 3E). The blue module membership had a high correlation (cor = 0.89, *P* value = 9.3e-177) with IRGs for Immunity_H (Fig. 3F). Thus, we chose blue module for next screening hub IRGs.

### 3.4. Hub IRGs screening by WGCNA and Cytoscape

**Figure 4.**
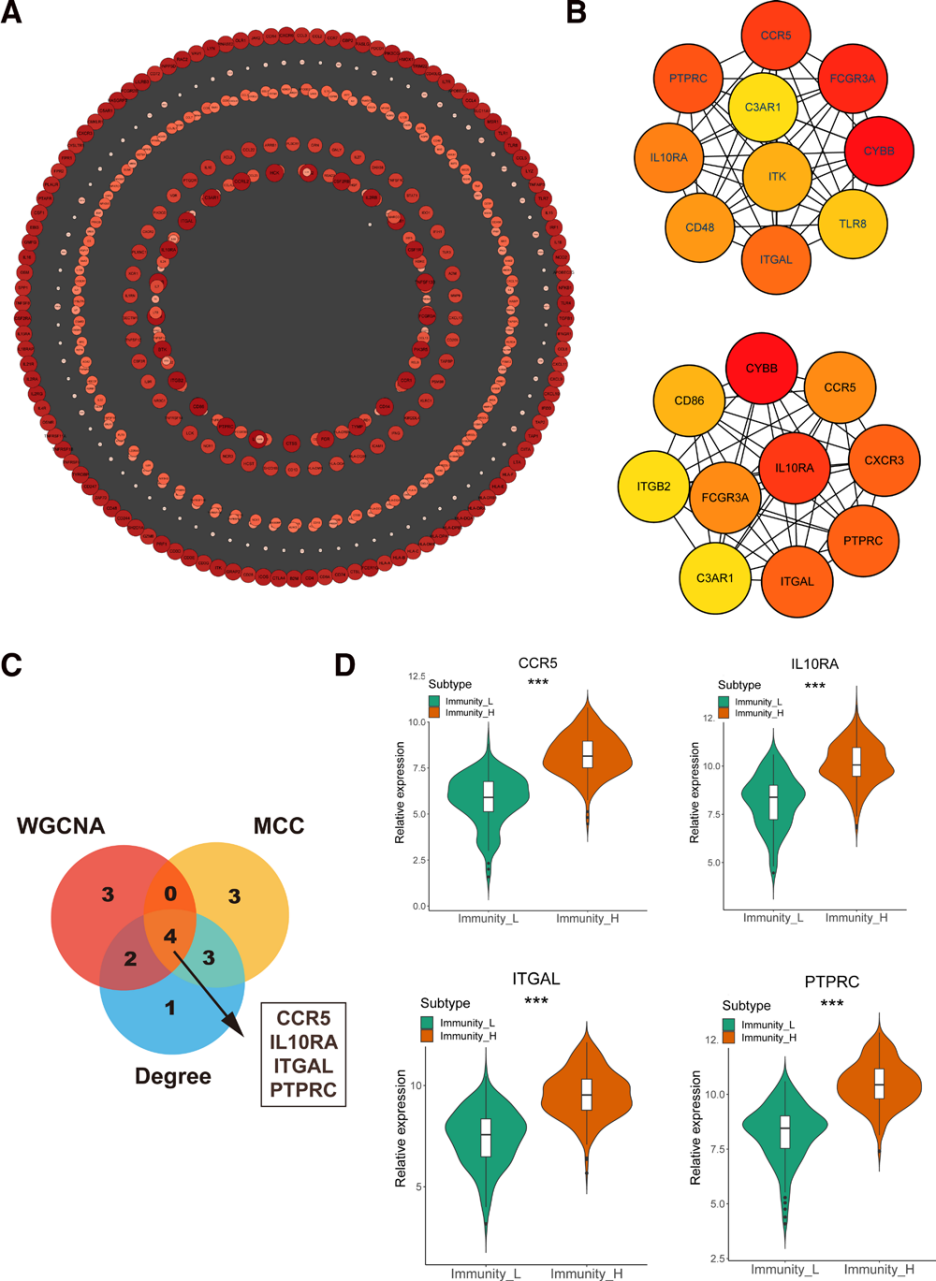
Identification of 4 hub IRGs of OV. (A) The PPI network construction of blue module. (B) Top 10 hub IRGs analyzed by cytoHubba (MCC and Degree methods). (C) Four hub IRGs (CCR5, IL10RA, ITGAL, and PTPRC) were intersected by Veen map. (D) The boxplot of 4 hub IRGs in 2 immune subtypes. IRGs = immune-related genes, OV = ovarian cancer, PPI = protein–protein interaction.

After blue module identified, we screened total number 9 hub genes (CCR5, CD86, BTK, IL10RA, IL12RB1, ITGAL, ITGB2, LCP2 and PTPRC) by setting up cor.geneModuleMembership > 0.9 and cor.geneTraitSignificance > 0.20. To validate the real hub IRGs, a total number of 462 IRGs in blue module were imported into Cytoscape for constructing network (Fig. 4A). At the same time, we used 2 methods (MCC and Degree) to screen top10 hub IRGs. The MCC method found top10 hub IRGs (CCR5, FCGR3A, CYBB, TLR8, ITGAL, CD48, IL10RA, PTPTC, C3AR1 and ITK). And the top10 hub IRGs, screened by Degree were CCR5, CXCR3, PTPRC, ITGAL, C3AR1, ITGB2, CD86, CYBB, FCGR3A and IL10RA (Fig. 4B). Four real hub IRGs (CCR5, IL10RA, ITGAL and PTPRC) were screened by intersecting these 3 methods (Fig. 4C). Interestingly, these 4 IRGs had higher expression in Immunity_H compared with Immunity_L (Fig. 4D).

### 3.5. Eight checkpoint genes expression and 4 hub IRGs mutation

**Figure 5.**
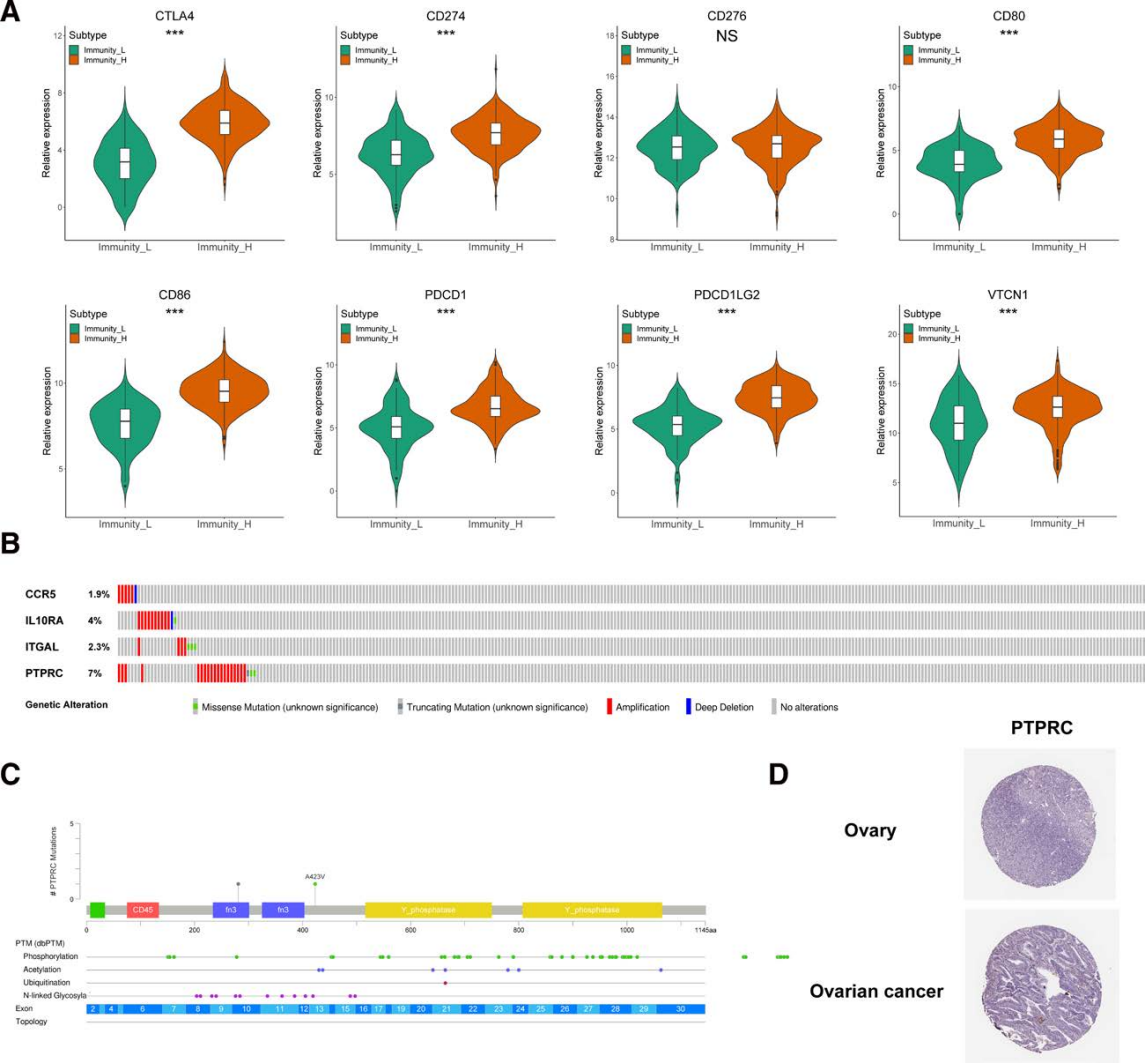
The extra exploration of 2 immune subtypes and 4 hub IRGs. (A) Eight immune checkpoint genes (CTLA4, CD274, CD276, CD80, CD86, PDCD1, PDCD1LG2, and VTCN1) expression between 2 immune subtypes. (B) The mutation information of 4 hub IRGs. (C) The detailed mutation site of PTPRC. (D) The immunohistochemical information of PTPRC in ovary and OV tissues. IRGs = immune-related genes, OV = ovarian cancer.

Immune checkpoints expression had influence on immunotherapy. Most of 8 key checkpoint genes (CTLA4, CD274, CD80, CD86, PDCD1, PDCD1LG2 and VTCN1) had higher expression in Immunity_H compared with Immunity_L, except CD276 (Fig. 5A). To explore further mechanism of these 4 hub IRGs, we searched cBioPortal online website. Apparently, PTPRC, also named CD45 had 7% amplifications and part missense mutations compared with the other hub IRGs (Fig. 5B). And the specific mutation sites of PTPRC were also explored (Fig. 5C). The immunohistochemical Information of PTPRC indicated PTPRC mostly located in nucleus and had higher expression in OV compared with ovary (Fig. 5D).

### 3.6. Correlation and GSEA analysis for 4 hub IRGs

**Figure 6.**
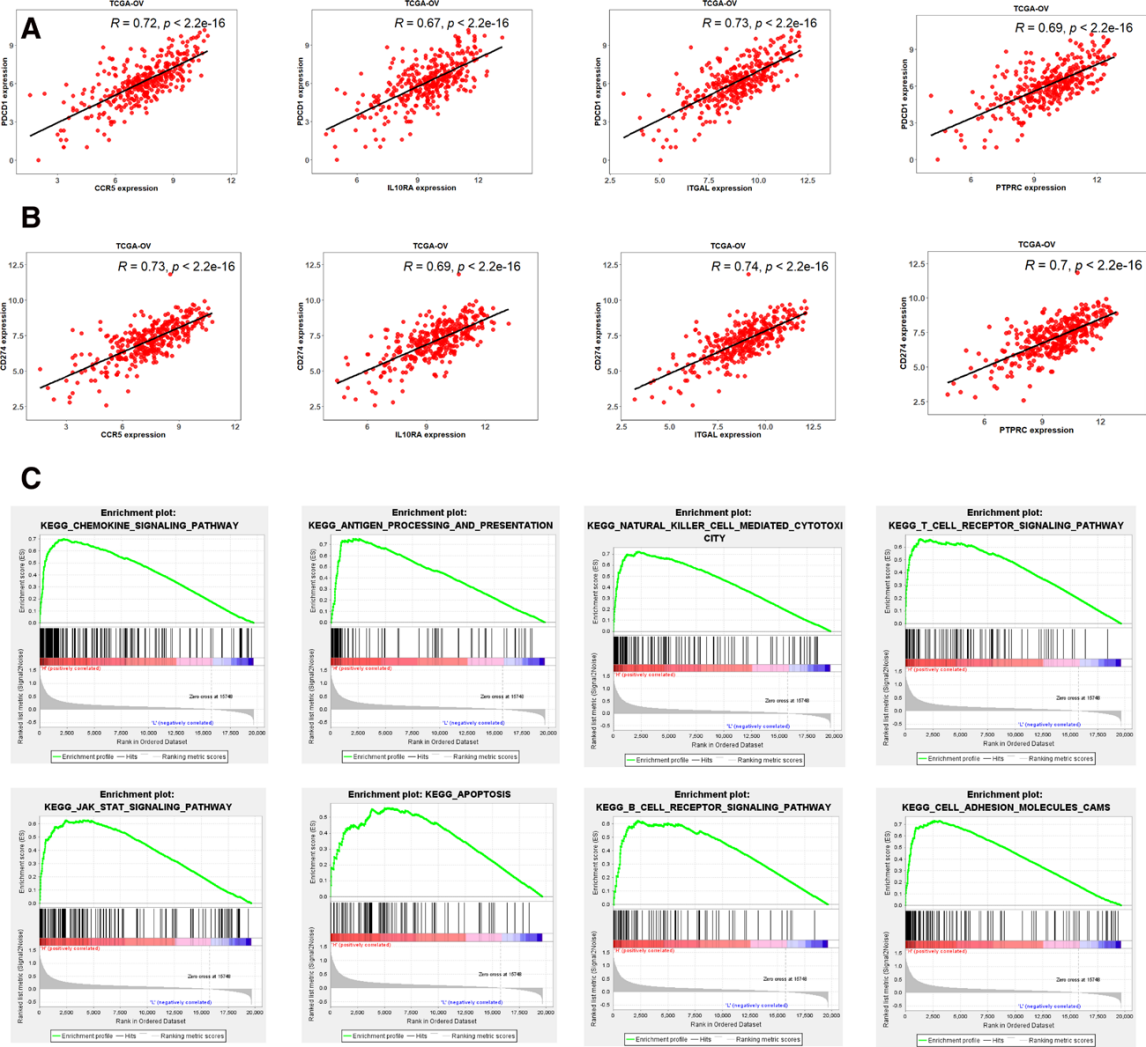
The correlation between 4 hub IRGs and PD-1/PD-L1 and GSEA for PTPRC. Four hub IRGs showed high correlation (>0.6) with (A) PD-1 and (B) PD-L1. (C) Plenty immune related signaling pathways were enriched by GSEA for PTPRC. IRGs = immune-related genes, GSEA = gene set enrichment analysis.

Besides, both the PD-1 (Fig. 6A) and PD-L1 (Fig. 6B) showed the extremely high correlation (*R* > 0.6) with these 4 hub IRGs. These results demonstrated these 4 hub IRGs had profound influence in immunomodulatory in OV. We found PTPRC had more amplification and mutation sites, which mean this hub IRGs might play vital role in immunomodulatory in OV. It`s urgent for us to explore the potential mechanisms of PTPRC by GSEA. There are many immune related pathways are enriched, like chemokine signaling pathway, antigen processing and presentation, natural killer cell mediated cytotoxicity, T cell receptor signaling pathway and B cell receptor signaling pathway (Fig. 6C). At the same time, conventional signaling pathways, like JAK STAT signaling pathway and apoptosis were also enriched (Fig. 6C). All these results revealed these 4 IRGs could promote progression of OV via multiple ways, especially in immune related pathways.

### 3.7. PTPRC promoted PD-L1 expression via JAK-STAT signaling pathway

**Figure 7.**
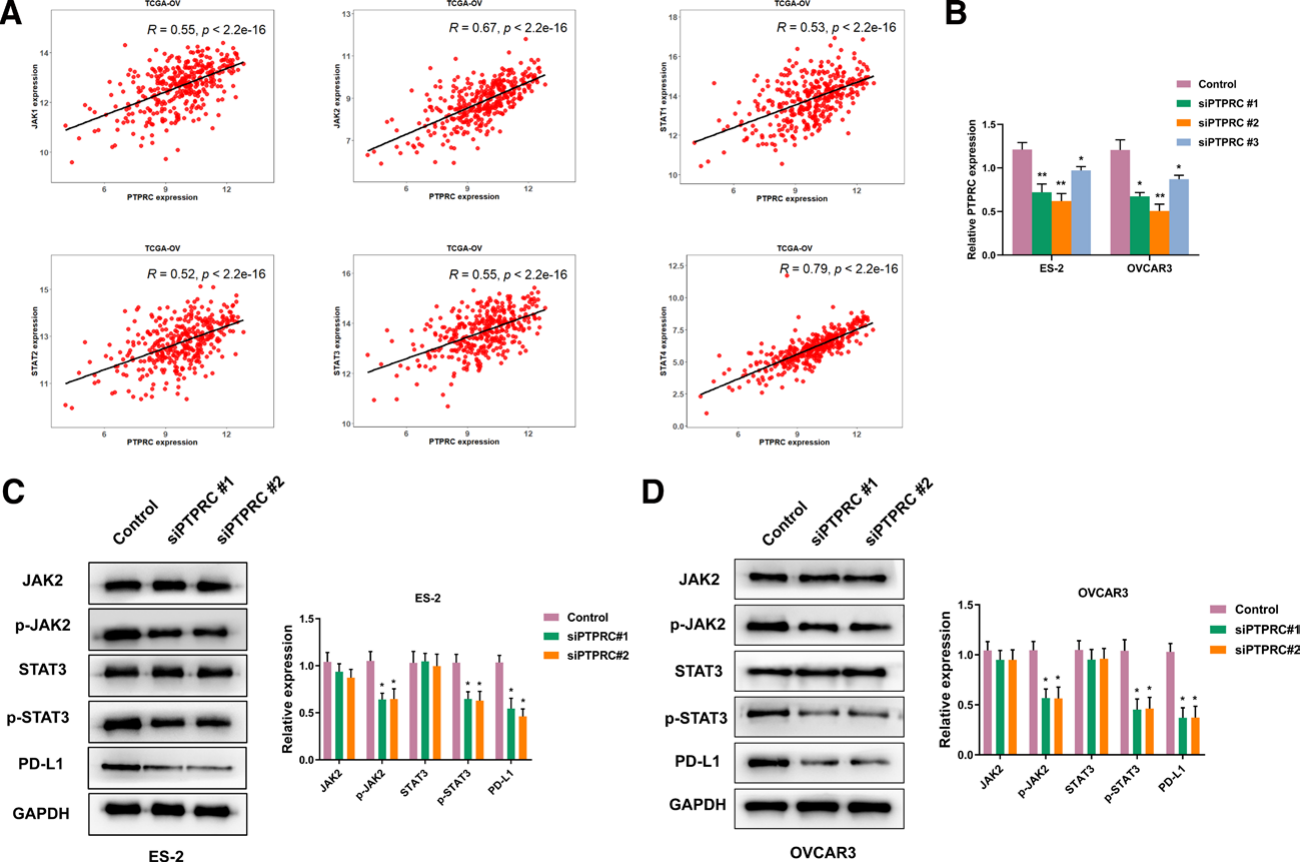
PTPRC promoted PD-L1 expression via JAK-STAT pathway. (A) The correlation of PTPRC with 6 hub genes in JAK-STAT signaling pathway. (B) The efficiency of knock down PTPRC in ES-2 and OVCAR3 2 OV cell lines. (C) The western blotting showed PD-L1 expression was influenced by JAK-STAT signaling pathway in ES-2 and OVCAR3. OV = ovarian cancer.

There are also JAK-STAT and apoptosis signaling pathways were enriched in OV by GSEA (Fig. 6C). Previous studies have showed PD-L1 expression was up-regulated by activating JAK-STAT signaling pathway in plenty tumors.^[27–29]^ However, whether PD-L1 was regulated by JAK-STAT in OV was rarely studied. Based on the preliminary study, our team had a shallower exploration for this molecular mechanism. Firstly, the key genes (JAK1, JAK2, STAT1, STAT2, STAT3, STAT4) in JAK-STAT signaling pathway had high correlation (*R* > 0.5) with PTPRC in OV (Fig. 7A). These results combined GSEA implied PTPRC might regulate JAK-STAT in OV. To validate this theoretical assumption, PTPRC expression was reduced using siRNAs in ES-2 and OVCAR3 cell lines. We chose siPTPRC #1 and #2 for next analysis (Fig. 7B). Then, we validated the phosphorylation of JAK2 and STAT3 was reduced in 2 siPTPRC groups compared with normal group. At the same time, the PD-L1 expression was also mitigated with PTPRC decline (Fig. 7C and D). Thus, we team hold an idea that PTPRC could regulate PD-L1 expression via JAK-STAT signaling pathway. This result might provide a new insight for immune therapy in OV.

## 4. Discussion

Advanced OV is an aggressive tumor which seriously harmed women’s health. Surgery and chemotherapy are the mainstream treatments for OV. Multi-disciplinary and multi-mode therapies of tumor is the trend of future development.^[30]^ Thus, we focused on immunotherapy exploration of OV by multiple bioinformatics methods, including ssGSEA, GSVA, WGCNA and so on.

Previously studies have disclosed plenty researches related to OV immune subtypes and immune microenvironments which implied the gradual rise of immune checkpoint inhibitors treatment of OV.^[31–34]^ The purpose of this study is to understand the immune landscape of OV. Our team found 2 immunity subtypes, which had influence on long-term overall survival for OV patients. And the Immunity_H had higher stromal score, immune score, and estimate score compared with Immunity_L subtype. The tumor purity had the adverse tendency. At the same time, among 29 immune gene sets, the Immunity_H subtype showed higher expression. All these findings indicated Immunity_H subtype had high correlation with immune status of OV microenvironment. Besides, the GSVA enrichment results showed positive relationship between improved immunoreaction and pathways correlated to classical signaling pathway (PI3K/AKT/MTOR, P53, TNFA/NFkB signaling pathways) and immune responses (T/B cell receptor signaling pathways and primary immunodeficiency). One review indicated that some hallmarks, like angiogenesis had synergistic effect with tumor micro-environment in OV.^[35]^ Though, anti-angiogenics (VEGF antibodies, tyrosine kinase inhibitors and angiopoietin antagonists) and polyadenosine diphosphate-ribose polymerase inhibitors are the most helpful drug treatment for OV. There are also other patterns target signaling pathways such as the PI3K/AKT/mTOR network and the epidermal growth factor receptor.^[36]^ The GSVA result also confirmed this truth. A total number of 4 IRGs were dug by 3 hub genes screening ways. One research indicated that C-C motif chemokine receptor 5 (CCR5) enhanced T-cell responses to tumors by modulating helper-dependent CD8(+) T-cell activation.^[37]^ It was reviewed CCR5 could promote immune responses by recruiting immune cells to the sites of inflammation/tumor which might act as a potential immune-checkpoint for cancers.^[38]^ One study emphasized mutations of Interleukin-10 receptor alpha (IL10RA), boosted early onset-inflammatory bowel disease occurrence.^[39]^ The role of IL10RA in anti-inflammatory response and immune response has been reported times during tumorigenesis.^[40–42]^ However, the elaborate mechanism of IL10RA in OV has not been reported yet. Aberrant expression of Integrin alpha L (ITGAL), a member of the integrin family, is related to tumorigenesis and immune response. And ITGAL could active multiple immune cells, which might regulate tumor immune microenvironment resulting in poor prognosis in gastric cancer.^[43]^ Our team expected more detailed mechanism of ITGAL to be explored in OV. The last hub IRG, PTPRC, also called CD45, is a member of the protein tyrosine phosphatase (PTP) family. PTPs are known to be signaling molecules that regulate a variety of cellular processes including cell growth, differentiation, mitosis, and oncogenic transformation. One research showed the sensitivity and specificity in detecting circulating tumor cells (CTCs) of OVs were improved by CD45-FISH compared with conventional immunocytochemistry staining-cytokeratin method.^[44]^ Interestingly, nearly 7% amplification of PTPRC in TCGA-OV samples might indicate deeper study value. All studies indicated these 4 IRGs might play a vital status in immune effect of OV. Besides, 8 hub immune checkpoint genes were also had significant expression tendency in 2 subtypes, except CD276. This result also verified the success of our immune subtypes. The high correlation between 4 IRGs and PD-1/PD-L1 and the GSEA results of PTPRC further emphasize these 4 IRGs promote progression of OV via multiple ways, especial immune related pathways. The PD-L1 expression was enhanced by activating JAK-STAT signaling pathway in multiple tumors.^[27–29]^ A shallow experiment was also verified in OV. All these results demonstrated that targeting PTPRC might a promising way for immune therapy in OV.

There are also part limitations in our current study. One limitation is we had no extra sequencing OV data to validate our present study. However, our team thought the TCGA-OV samples were plenty enough to support our results. Another limitation is that more elaborate immune experiments should be conducted for these 4 hub IRGs, especially PTPRC. Although, our team conducted cursory experiment for PTPRC in OV. As far as we are concerned, PTPRC might have great potential in the field of OV immunotherapy.

## 5. Conclusion

In a word, our study identified 2 immune subtypes of OV which might play another promising new approach against OV. The hallmarks and Kyoto Encyclopedia of Genes and Genomes pathways in 2 immune subtypes were also explored by GSVA method. Meanwhile, a total number of 4 IRGs were dug by WGCNA and Cytoscape and we explored the mutations of 4 IRGs. Finally, the high correlation between 4 IRGs and PD-1/PD-L1 and the GSEA results of PTPRC further emphasized these 4 IRGs might promote progression of OV through multiple ways, especial immune related pathways. Our team also validated PTPRC regulated PD-L1 expression via activating JAK-STAT signaling pathway. All these results might provide novel horizon for OV immune therapy.

## Acknowledgements

Our team would show great gratitude for all participants.

## Author contributions

**Conceptualization:** Qin Tang, Haojie Zhang, Rong Tang.

**Data curation:** Qin Tang, Haojie Zhang, Rong Tang.

**Formal analysis:** Qin Tang, Rong Tang.

**Software:** Rong Tang.

**Validation:** Rong Tang.

## Supplementary Material
