# Supplementary material for: Identification of two immune subtypes and four hub immune-related genes in ovarian cancer through multiple analysis
Source: Medicine (Baltimore). 2023 Oct 6;102(40):e35246. doi: 10.1097/MD.0000000000035246 (PMC10553066; doi:10.1097/MD.0000000000035246)
Supplement: Supplementary file 1 [file medi-102-e35246-s001.docx]

The siRNAs sequence of PTPRC.

| **Gene sybmol** | **siRNA type** | **Sequence (from 5` to 3`)** |
| --- | --- | --- |
| siPTPRC#1 | Sense | GCAGGGUCAAACUACAUAAAUTT |
|  | Anti-sense | AUUUAUGUAGUUUGACCCUGCTT |
| siPTPRC#2 | Sense | GCUGCACAUCAAGGAGUAAUUTT |
|  | Anti-sense | AAUUACUCCUUGAUGUGCAGCTT |
| siPTPRC#3 | Sense | CCAGACAAUACUUCCACCCAATT |
|  | Anti-sense | UUGGGUGGAAGUAUUGUCUGGTT |
| Control | Negative control | UUCUCCGAACGUGUCACGU(dT)(dT) |

The antibody information.

| **Antibody** | **Company** | **Type** | **WB** |
| --- | --- | --- | --- |
| JAK2(#3230) | CST | Rabbit monoclonal | 1:1000 |
| p-JAK2(#66245) | CST | Rabbit monoclonal | 1:1000 |
| STAT3(#12640) | CST | Rabbit monoclonal | 1:1000 |
| p-STAT3(#9145) | CST | Rabbit monoclonal | 1:1000 |
| PD-L1(#13684) | CST | Rabbit monoclonal | 1:1000 |
| GAPDH(#5174) | CST | Rabbit monoclonal | 1:1000 |
